# Transcriptome analysis in LRRK2 and idiopathic Parkinson’s disease at different glucose levels

**DOI:** 10.1038/s41531-021-00255-x

**Published:** 2021-12-01

**Authors:** Rubén Fernández-Santiago, Anna Esteve-Codina, Manel Fernández, Francesc Valldeoriola, Almudena Sanchez-Gómez, Esteban Muñoz, Yaroslau Compta, Eduardo Tolosa, Mario Ezquerra, María J. Martí

**Affiliations:** 1 Lab of Parkinson disease and Other Neurodegenerative Movement Disorders: Clinical and Experimental Research, Department of Neurology, Hospital Clínic of Barcelona, Institut d’Investigacions Biomèdiques August Pi i Sunyer (IDIBAPS), Institut de Neurociències, Universitat de Barcelona, 08036 Barcelona, Catalonia Spain; 2 Centro de Investigación Biomédica en Red de Enfermedades Neurodegenerativas (CIBERNED), 08036 Barcelona, Catalonia Spain; 3 CNAG-CRG, Centre for Genomic Regulation, Barcelona Institute of Science and Technology, 08028 Barcelona, Catalonia Spain; 4 Universitat Pompeu Fabra (UPF), Barcelona, Catalonia Spain; 5 Parkinson’s disease & Movement Disorders Unit, Neurology Service, Hospital Clínic de Barcelona, Institut d’Investigacions Biomèdiques August Pi i Sunyer (IDIBAPS), University of Barcelona, 08036 Barcelona, Catalonia Spain

**Keywords:** Parkinson's disease, Gene expression profiling

## Abstract

Type-2 diabetes (T2D) and glucose metabolic imbalances have been linked to neurodegenerative diseases, including Parkinson’s disease (PD). To detect potential effects of different glucose levels on gene expression, by RNA-seq we analyzed the transcriptome of dermal fibroblasts from idiopathic PD (iPD) patients, LRRK2-associated PD (L2PD) patients, and healthy controls (total *n* = 21 cell lines), which were cultured at two different glucose concentrations (25 and 5 mM glucose). In PD patients we identified differentially expressed genes (DEGs) that were related to biological processes mainly involving the plasmatic cell membrane, the extracellular matrix, and also neuronal functions. Such pathway deregulation was largely similar in iPD or L2PD fibroblasts. Overall, the gene expression changes detected in this study were associated with PD independently of glucose concentration.

## Introduction

Parkinson’s disease (PD) is a neurodegenerative disorder associated with the progressive loss of dopaminergic neurons (DAn) in the substantia nigra. Although most patients are idiopathic PD cases (iPD) of unknown cause, around 5–10% encompass monogenic forms caused by pathogenic mutations. Among these, mutations in the leucine-rich repeat kinase 2 gene (*LRRK2*) causing *LRRK2*-associated PD (L2PD) are the most frequent cause of autosomal-dominant PD but also of sporadic cases by reduced penetrance^1^. Clinically, L2PD largely resembles iPD, suggesting that both PD types could share similar pathogenic mechanisms^2^. The most frequent *LRRK2* G2019S mutation has been associated with cytoskeletal remodeling and abnormal vesicle trafficking resulting in shortening of the axonic and dendritic trees in cultured neurons^3^.

Imbalances of glucose metabolism and type-2 diabetes (T2D) have been linked to PD, eventually with conflicting results^4,5^. A recent meta‐analyses of observational studies and genetic data showed compelling evidence supporting an effect of diabetes on PD risk^6^. In fact, a certain degree of molecular pathway overlap between PD and T2D has been reported^7,8^. Yet, studies addressing specific effects of glucose levels in PD patients are needed. In this context, in the present study, we explored the transcriptome of PD by comprehensive RNA-seq analyses using primary skin fibroblasts from patients with iPD and monogenic L2PD, which were cultured at low and high-glucose concentrations (5 vs. 25 mM glucose). The goals of the study were to assess differential gene expression changes associated with PD using peripheral fibroblasts cells, compare the transcriptome of the iPD and L2PD disease forms, and explore the potential effects of different culture glucose levels on the PD transcriptome.

## Results

### Transcriptomic analysis in the discovery cohort

**Fig. 1:**
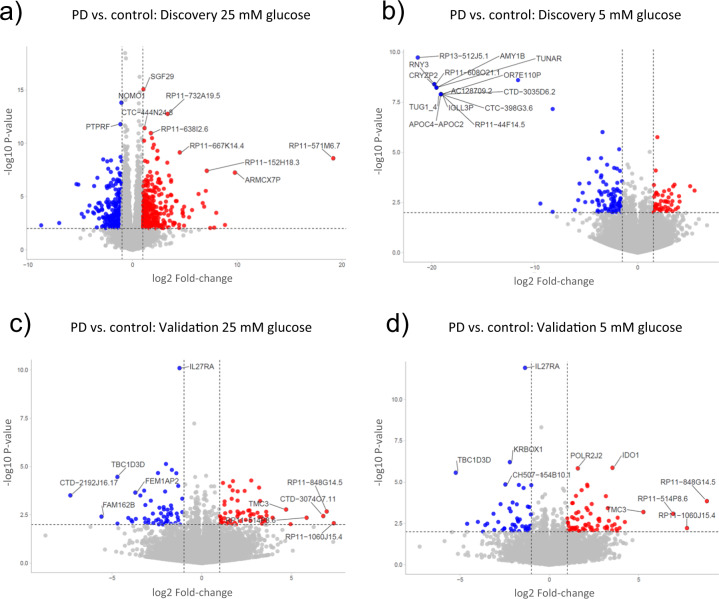
Volcano plots showing gene expression differences between overall PD and controls across different comparisons. Differentially expressed genes (DEGs) with a *P*-value <0.05 and a fold-change FC ≥ |2| are depicted in red (up-regulated) and blue (down-regulated). **a** Discovery cohort at 25 mM glucose. **b** Discovery cohort at 5 mM glucose. **c** Validation cohort at 25 mM glucose. **d** Validation cohort at 5 mM glucose. Volcano plots were done using default settings at the VolcaNoSer software. Annotated dots correspond to the ten DEG data points with the largest (Manhattan) distance from the origin above the significance thresholds indicated by the dashed line. VolcaNoSer: https://huygens.science.uva.nl/VolcaNoseR/.

**Fig. 2:**
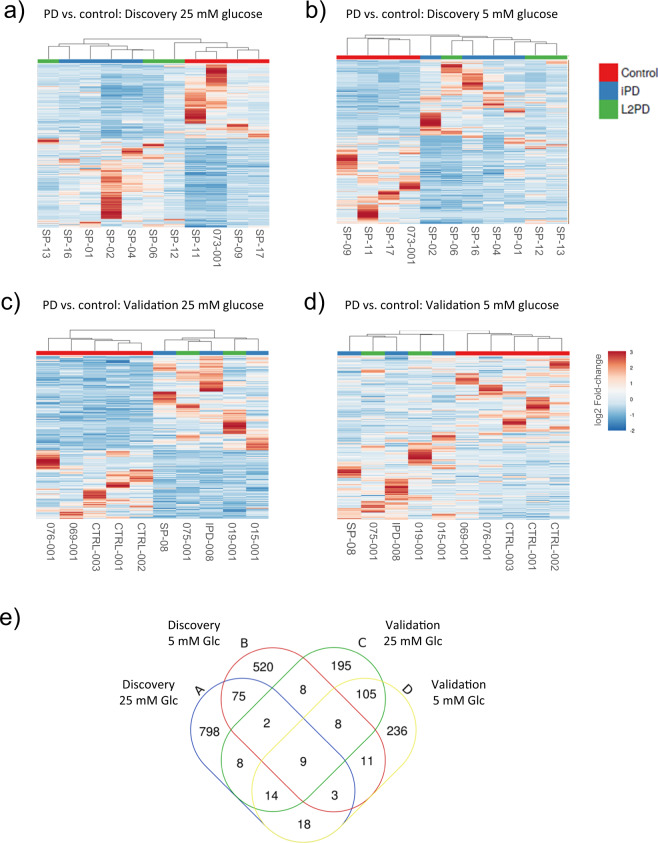
Hierarchical clustering analysis of L2PD patients, iPD patients, and controls with heatmap density color representation of differentially expressed genes (DEGs) across different comparisons. **a** Discovery cohort at 25 mM glucose. **b** Discovery cohort at 5 mM glucose. **c** Validation cohort at 25 mM glucose. **d** Validation cohort at 5 mM glucose. Heatmaps of DEGs (*P*-value<0.05 and fold change FC ≥ |2|) were performed using default settings at the Clustvis software. **e** Venn diagram representation showing DEG overlap between PD vs. controls in the four different comparisons, where “A” corresponds to discovery 25 mM glucose, “B” discovery 5 mM glucose, “C” Validation 25 mM glucose, and “D” validation 5 mM glucose. Clustvis: https://biit.cs.ut.ee/clustvis/.

In the discovery phase, at a low-glucose concentration medium (5 mM Glucose), we found 636 DEGs associated with disease in the overall PD group compared to healthy controls (Fig. 1a, b). Segregating by subtype, we found 846 DEGs in iPD and 594 in L2PD (Supplementary Table 1). At high-glucose medium (25 mM Glucose), we identified 931 DEGs in the whole PD group with respect to controls and, by subtype, 1295 DEGs in iPD and 664 in L2PD. In addition, hierarchical clustering analysis of gene expression data confirmed a highly similar expression profile in all PD cases for which L2PD and iPD clustered together apart from controls in both low and high-glucose conditions (Fig. 2a, b).

### Transcriptomic analysis in the validation cohort

In addition, we used an independent set of additional iPD and L2PD patients to assess the validity of these results. Compared to controls, in the overall PD group, we again obtained similar transcriptomic changes with 405 DEGs associated with PD at low glucose (484 DEGs in iPD and 339 in L2PD), and 350 DEGs associated with PD at high-glucose (369 DEGs in iPD and 302 in L2PD) (Fig. 1c, d and Supplementary Table 2). As in the discovery set, hierarchical clustering analysis of DEGs at the validation phase also revealed similar expression patterns in iPD and L2PD with respect to controls both in high and low glucose (Fig. 2c, d)

### Biological enrichment analysis

To assess the biological functions of the identified PD-associated DEGs at low and high-glucose concentration, we performed a functional gene enrichment analysis. In the discovery set, we observed a statistically significant association with biological processes mainly related to the plasmatic membrane, cell adhesion, extracellular matrix, and neuronal processes (Table 1). Despite the higher number of DEGs found in iPD with respect to L2PD which are potentially related to the higher number of iPD studied patients than L2PD, we found very similar affected biological terms in iPD or L2PD compared separately with controls (Supplementary Table 3). In the validation set, the biological enrichment analysis at low and high glucose again revealed biological terms mainly related to the extracellular matrix, cell adhesion, plasma membrane, and synapsis (Table 1 and Supplementary Table 3). Remarkably, despite the limited DEG overlap across all four comparisons (Fig. 1e) we found a highly consistent pathway deregulation (Table 1 and Supplementary Table 3).

**Table 1:** Biological enrichment analysis in all PD vs. controls.

Discovery PD at 25 mM glucose			Discovery PD at 5 mM glucose		
*Biological process*	*P-value*	*Adj P-value*	*Biological process*	*P-value*	*Adj P-value*
Cell adhesion	9.85E−31	2.84E−27	Signal transduction	1.26E−19	2.82E−16
Homophilic cell adhesion via plasma membrane adhesion	8.42E−17	1.22E−13	G protein-coupled receptor signaling pathway	4.51E−17	5.06E−14
Nervous system development	2.91E−16	2.80E−13	Multicellular organism development	2.20E−15	1.64E−12
Extracellular matrix organization	1.19E−15	8.55E−13	Cell adhesion	2.64E−14	1.48E−11
Multicellular organism development	1.65E−15	9.54E−13	Positive regulation of ERK1 and ERK2 cascade	1.43E−10	6.40E−08
*Cell component*	*P-value*	*Adj P-value*	*Cell component*	*P-value*	*Adj P-value*
Membrane	1.67E−70	7.83E−68	Integral component of membrane	4.31E−54	1.61E−51
Integral component of membrane	8.47E−69	1.99E−66	Membrane	6.59E−50	1.24E−47
Plasma membrane	3.46E−56	5.42E−54	Plasma membrane	4.86E−35	6.07E−33
Extracellular region	5.86E−45	6.88E−43	Extracellular region	4.03E−34	3.78E−32
Cytoplasm	6.59E−40	6.20E−38	Cytoplasm	1.14E−25	8.56E−24

Validation PD at 25 mM glucose			Validation PD at 5 mM glucose		
*Biological process*	*P*-*value*	*Adj* *P*-*value*	*Biological process*	*P-value*	*Adj P-value*
Cell adhesion	7.86E−09	9.62E−06	Synapse assembly	6.56E−08	7.57E−05
Synapse assembly	2.32E−08	1.42E−05	Cell adhesion	1.94E-06	0.0011
Ion transmembrane transport	1.31E−07	5.34E−05	Adult behavior	3.75E-05	0.0054
Multicellular organism development	4.02E−07	0.0001	G protein-coupled receptor signaling pathway	4.62E-05	0.0059
Immune system process	5.58E−07	0.0001	Signal transduction	3.63E-05	0.0059
*Cell component*	*P*-*value*	*Adj* *P*-*value*	*Cell component*	*P*-*value*	*Adj P-value*
Membrane	7.71E−29	1.80E−26	Plasma membrane	2.11E−23	5.12E−21
Integral component of membrane	2.39E−26	2.78E−24	Membrane	6.00E−21	7.26E−19
Plasma membrane	5.07E−26	3.94E−24	Integral component of membrane	7.91E−18	4.78E−16
Extracellular region	2.45E−21	1.43E−19	Extracellular region	6.12E−18	4.94E−16
Extracellular space	1.81E−15	8.46E−14	Extracellular space	2.70E−14	1.31E−12

Top gene ontology (GO) terms of genes associated with differentially expressed transcripts in the discovery and validation studies identified in fibroblasts from all PD patients (iPD and L2PD) as compared to controls independently at high (25 mM) and low (5mM) glucose conditions. *P*-values were adjusted by false discovery rate (FDR) multiple testing correction.

## Discussion

Here we report transcriptome alterations in primary skin fibroblast from a set of patients with iPD and monogenic L2PD for the first time. Akin to previous observations showing that L2PD is clinically undistinguishable from iPD^1,2^, here we found that the functional pathways altered at the transcriptome level in iPD and L2PD are also largely similar, thus indicating similarities at the molecular level between both disease forms. Yet, these results should be interpreted with caution considering the reduced number of samples when segregating PD patients into iPD and L2PD. Although we found a limited gene overlap among all four comparisons, we also found that biological pathways affected in PD patients, either L2PD or iPD, were very similar across the different cohorts and comparisons. These gene expression changes were independent of different glucose concentrations under the tested culture conditions.

The identified expression changes associated with PD in idiopathic and monogenic L2PD involved alterations related to the plasma membrane, extracellular matrix, cell adhesion, and neural processes. These results are in line with previous studies reporting deregulation in the cytoskeleton, cell adhesion, or extracellular matrix processes in various PD patients biospecimens such as blood cells^9,10^, iPSC-derived DAn^11,12^, or post-mortem substantia nigra^13^. Specifically, the previous transcriptomic study available in PD fibroblasts was done in patients carrying *PRKN* mutations^14^. Interestingly, *PRKN*-associated PD showed deregulation of cell adhesion processes and other terms that emerged specific for this disease form, such as aminoacid and folate metabolism suggesting the presence of additional alterations in this other PD monogenic. In addition, a remarkable finding is that given the peripheral non-neuronal type cell nature of skin fibroblasts, we found biological enrichment in terms related to neuron or synapsis in iPD and L2PD. These results can be interpreted given that neurons and non-neuronal peripheral cells such as fibroblasts share common molecular mechanisms involving cell adhesion and extracellular matrix leading to filopodia and cilia formation^15^, which mediate axon guidance and synaptic plasticity in a neural cell context^16^.

Overall, PD fibroblasts have been mostly used to investigate PD mitochondrial phenotypes until date^17,18^and to study cytoskeleton and cell adhesion regulatory mechanisms^19^. Based on our findings that the main expression changes detected in iPD and L2PD fibroblasts are related to cytoskeleton rather than mitochondria, additional functional studies investigating the interplay between gene expression deregulation and cytoskeleton-related processes are warranted in PD fibroblasts, most especially given the easy availability and robustness of this cell model. In addition, some of the top hits across all comparisons (Fig. 1) have been previously linked to PD. For example, the APOC4-APOC2 protein was significantly altered in plasma from PD patients^20^ and NOMO1 in isolated neurons from post-mortem sustantia nigra of PD patients^21^. Moreover, TUG1 was shown to modify neurodegeneration in a cell model of PD^22^, and IDO1 in a PD animal model^23^.

Previous studies suggested a role for glucose imbalances in PD pathology, including an association of T2D with PD, as reported in a recent comprehensive meta-analysis^6^. In PD fibroblasts, some studies reported alterations in the glycolytic activity and that this glycolytic capacity can be altered by the presence or absence of media glucose, although results are still conflicting and need further research (reviewed in ref. ^24^). In our study we did not observe an influence of different glucose concentrations on the biological processes related to PD, at least at the transcriptomic level and under the tested culture conditions. However, we cannot discard that glucose imbalances in the studied PD fibroblasts could affect other processes beyond gene expression like metabolic and mitochondrial plasticity^25^. Indeed, other studies described cell death effects of extracellular glucose levels in PD dopaminergic neurons^26^.

In summary, we identified similar biological processes altered in the transcriptome from iPD and G2019S L2PD related to cell adhesion processes and neural functions. These biological processes were associated with PD but did not seem to be influenced by the studied glucose concentrations in our cell model.

## Methods

### Subjects

Primary skin biopsies were obtained from iPD patients without a family history of disease and without *LRRK2* mutations, L2PD patients carrying the G2019S mutation, and cultured fresh. The resulting dermal fibroblasts were expanded and cryopreserved in liquid N_2_ until use. In the discovery cohort, we screened seven PD patients (four iPD patients and three unrelated L2PD) and four healthy controls with no history of neurological disease. The validation cohort included five PD patients (three iPD and two unrelated L2PD) and five controls (Table 2). All subjects were Europeans of Spanish origin. We used Taqman assays (Thermo Fisher Sci.) on a Step One Plus Real-time PCR System (Thermo Fisher Sci.) to genotype LRRK2 G2019S (#C-63498123-10), and another TaqMan assay to genotype R1441G/C/H as previously described^27^. All 5 L2PD patients at discovery and validation were heterozygous G2019S carriers. Of these, 4 L2PD patients were familial cases with another affected family member, and 1 L2PD was the only affected family member. The studied LRRK2 mutations were absent in the iPD patients and healthy controls. No systematic sequencing was done to exclude additional mutations causing monogenic PD. The study was approved by the local ethics committee and conducted according to the Declaration of Helsinki. Written informed consent was obtained from all participating subjects. Patient data were encoded to protect confidentiality.

**Table 2:** Summary of subject clinical features from Parkinson’s disease (PD) patients and controls.

Code	PD type	Familial PD	Affected members^a^	Gender	AAD	AAO	Initial Symptom^b^	L-DOPA response
*Discovery*								
SP-01	iPD	No	0	Female	63	58	T and B	NA
SP-02	iPD	No	0	Male	55	48	T	NA
SP-04	iPD	No	0	Male	46	40	B	Good
SP-06	L2PD	Yes	2	Male	44	33	T	Good
SP-12	L2PD	Yes	2	Female	63	49	T	Good
SP-13	L2PD	Yes	2	Female	68	57	T	Good
SP-16	iPD	No	0	Female	51	48	B	NA
SP-09	Control	No	0	Male	66	–	–	–
SP-11	Control	No	0	Female	48	–	–	–
SP-17	Control	No	0	Male	52	–	–	–
73001	Control	No	0	Female	66	–	–	–
*Validation*								
iPD008	iPD	No	0	Female	65	57	T	Good
SP08	iPD	No	0	Female	66	60	NA	NA
015-001	iPD	No	0	Male	68	56	T	Good
019-001	L2PD	Yes	2	Female	71	58	T	Good
075-001	L2PD	No	0	Male	51	47	B	Good
CTRL-001	Control	No	0	Male	68	–	–	–
CTRL-002	Control	No	0	Female	73	–	–	–
CTRL-003	Control	No	0	Female	55	–	–	–
069-001	Control	No	0	Male	64	–	–	–
076-001	Control	No	0	Female	61	–	–	–

^a^PD affected family members including the proband; AAD: age-at-donation, AAO: Age-at-onset.

^b^Initial symptoms (T, tremor; B, bradykinesia); L2PD, LRRK2-associated PD; iPD, idiopathic PD; NA, not assessed.

### Cell culture

Fibroblasts from PD patients and controls were simultaneously cultured per duplicate in DMEM high-glucose (25 mM) (Thermofisher #41966029), and DMEM low-glucose (5 mM) (Thermofisher #31885023), supplemented with 10% calf serum (Gibco #10500064), and penicillin 100 U/mL / streptomycin 100 μg/mL (Sigma #15140122) at 37 °C and 10% CO_2_. Fibroblasts were seeded at cell passage numbers between 2 and 6 for discovery and 1–3 for validation, grown until reaching the first confluence and passed using trypsin/EDTA (Sigma #15090046). After the second confluence, fibroblasts were harvested, and dry pellets were stored at −80 °C until subsequent RNA isolation using the AllPrep DNA/RNA/Protein kit (Qiagen #80004). The total culture period was of 10–14 days depending on the cell line. During this period, cells were constantly maintained in their corresponding glucose concentration medium, with regular medium renovation every 2–3 days. Regarding glucose conditions, normal fasting glucose level in the blood for non-diabetics is 3.9–5.6 mmol/L, and accordingly, 5 mM glucose represents a normoglycaemic condition. In addition, 25 mM glucose has been usually chosen in the scientific literature to model hyperglycemia in different cell culture types^28,29^, including PD fibroblasts where hyperglycaemic associated changes were reported^25^.

### mRNA library preparation and RNA-seq

Total RNA samples were quality controlled by Qubit^®^ RNA BR Assay kit (Thermo Fisher Scientific) for quantity and RNA 6000 Nano Assay (Agilent) for integrity. Samples were analyzed by RNA-seq in three different array batches as follows, including discovery batches 2 (high glucose) and batch 3 (low glucose), and validation batch 3 (high and low glucose).

Batch_02: The RNASeq libraries were prepared from total RNA using the TruSeq™ RNA Sample Prep Kit v2 (Illumina) with minor modifications. Briefly, after poly-A based mRNA enrichment with oligo-dT magnetic beads and 0.5 μg of total RNA as the input material the mRNA was fragmented. After first and second-strand cDNA synthesis the double-stranded cDNA was end-repaired, 3´adenylated and the Illumina barcoded adapters were ligated. The ligation product was enriched by 10 cycles of PCR. The final library was validated on an Agilent 2100 Bioanalyzer with the DNA 7500 assay. Each library was sequenced on HiSeq2000 (Illumina) in paired-end mode with a read length of 2 × 76 bp using TruSeq SBS Kit v4 in a fraction of a sequencing v4 flow cell lane, following the manufacturer’s protocol. Image analysis, base calling, and quality scoring of the run were processed using the manufacturer’s software Real Time Analysis (RTA 1.18.61) and followed by generation of FASTQ sequence files by CASAVA.

Batch_03: The RNASeq libraries were prepared from total RNA using the TruSeq™ Stranded mRNA Library Prep Kit (Illumina). In brief, the mRNA was enriched with oligo-dT magnetic beads from total RNA (500 ng) and it was fragmented to 80–450 nt. The second strand cDNA synthesis was performed in the presence of dUTP to achieve the strand specificity. The blunt-ended double-stranded cDNA was 3´adenylated and Illumina single-index adapters were ligated. The ligation product was enriched with 15 PCR cycles. The final library was validated on an Agilent 2100 Bioanalyzer with the DNA 7500 assay. The libraries were sequenced on HiSeq2000 (Illumina) in paired-end mode with a read length of 2x76bp using TruSeq SBS Kit v4 in a fraction of a sequencing v4 flow cell lane, following the manufacturer’s protocol. Image analysis, base calling and quality scoring of the run were processed using the manufacturer’s software Real Time Analysis (RTA 1.18.66.3) and followed by the generation of FASTQ sequence files.

Batch_04: The RNASeq libraries were prepared with KAPA Stranded mRNA-Seq Illumina® Platforms Kit (Roche) following the manufacturer´s recommendations. Briefly, 500 ng of total RNA was used as the input material, the poly-A fraction was enriched with oligo-dT magnetic beads and the mRNA was fragmented. The strand specificity was achieved during the second strand synthesis performed in the presence of dUTP instead of dTTP. The blunt-ended double-stranded cDNA was 3´adenylated and Illumina platform compatible adaptors with unique dual indexes and unique molecular identifiers (Integrated DNA Technologies) were ligated. The ligation product was enriched with 15 PCR cycles and the final library was validated on an Agilent 2100 Bioanalyzer with the DNA 7500 assay. The libraries were sequenced on HiSeq4000 (Illumina) in a fraction of a HiSeq 4000 PE Cluster kit sequencing flow cell lane, following the manufacturer’s protocol for dual indexing. Image analysis, base calling, and quality scoring of the run were processed using the manufacturer’s software Real Time Analysis (RTA 2.7.7) and followed by the generation of FASTQ sequence files.

### RNA-seq data processing and analysis

RNAseq reads were mapped against the human reference genome GRCh38 using STAR software version 2.5.1b^30^ with ENCODE parameters. Genes were quantified using RSEM version 1.2.28^31^with default parameters and annotation file from GENCODE version 24. Differential expression analysis was performed with DESeq2 v1.18. R package^32^ adjusting by sex and using the Wald test to compare the experimental conditions. We defined differentially expressed genes (DEGs) as those with P-value<0.05 and absolute fold-change difference |FC| > 2. To this end, we followed the cut-off consensus guidelines recommended for transcriptome studies in the clinical setting^33,34^ which have been specifically proposed for functional enrichment analysis^35^. Hierarchical clustering analyses were performed using the Clustvis software following the default standard settings (https://biit.cs.ut.ee/clustvis/). We computed Volcano plots showing fold-change and P-value distribution of all genes and DEG in the different comparisons (Fig. 1).

### RT-qPCR validation

For technical validation of the RNA-seq study, we performed a real-time quantitative PCR (RT-qPCR) analysis. To this end, we selected 9 among the top-deregulated DEGs from the RNA-seq 25 mM glucose discovery analysis and quantified their expression by RT-qPCR. Briefly, fibroblast cDNA was synthesized from 2 μg of total RNA using a high-capacity reverse transcription kit according to the manufacturer’s protocol (Thermo Fisher Scientific Inc. #4368813); and diluted 1/10 in RNase-free water. For amplification, we used the Taqman assays below (Applied Biosystems) in a StepOnePlus Real-Time PCR System applying the thermal cycling conditions indicated by the manufacturer (Thermo Fisher Scientific Inc.). Optimal results were obtained using 12.5 μL of TaqMan Gene Expression Master Mix (Thermo Fisher Scientific Inc. #4369514), 1.25 μL of TaqMan Gene Expression Assay, 8.75 μL of RNase-free water, and 2.5 μL of the diluted template cDNA in a final reaction volume of 25 μL in 96-well plates. For relative quantification, we applied the comparative threshold cycle (CT) method (ΔΔCt) using the Dataassist 3.0 software (Applied biosystems). The internal references or housekeeping genes included: *GAPDH* (Hs02758991_g1), *HPRT1* (Hs02800695_m1), and *PPIA* (Hs04194521_s1). The target genes included: *EGR1* (Hs00152928_m1)*, IGF2BP3* (Hs00559907_g1)*, ITGA4* (Hs00168433_m1)*, ANK3* (Hs00241738_m1)*, ITGA10* (Hs00174623_m1)*, MFAP4* (Hs00412974_m1)*, MYL3* (Hs00264820_m1), and *TMOD1* (Hs00984439_m1). Overall, we found a Pearson fold-change correlation coefficient of 0.95 (P-value=0.00019) between the RNA-seq and RT-qPCR (Supplementary Fig. 1).

### Functional enrichment analysis

To determine whether DEGs in PD were enriched in particular gene ontology (GO) terms, specifically in Biological process and Cellular component terms, we used the GeneCodis 4.0 software (https://genecodis.genyo.es/).

### Reporting summary

Further information on research design is available in the Nature Research Reporting Summary linked to this article.

## Supplementary information

Supplementary Information

Supplementary Data 1

Supplementary Data 2

Supplementary Data 3

Reporting Summary

## Data Availability

GEO data of the study and study details are deposited in the publicly available repositories GSE82340 and GSE167529.
