# Supplementary material for: Transcriptome analysis in LRRK2 and idiopathic Parkinson’s disease at different glucose levels
Source: NPJ Parkinsons Dis. 2021 Dec 1;7:109. doi: 10.1038/s41531-021-00255-x (PMC8636510; doi:10.1038/s41531-021-00255-x)
Supplement: Supplementary file 1 — Supplementary Information [file 41531_2021_255_MOESM1_ESM.pdf]

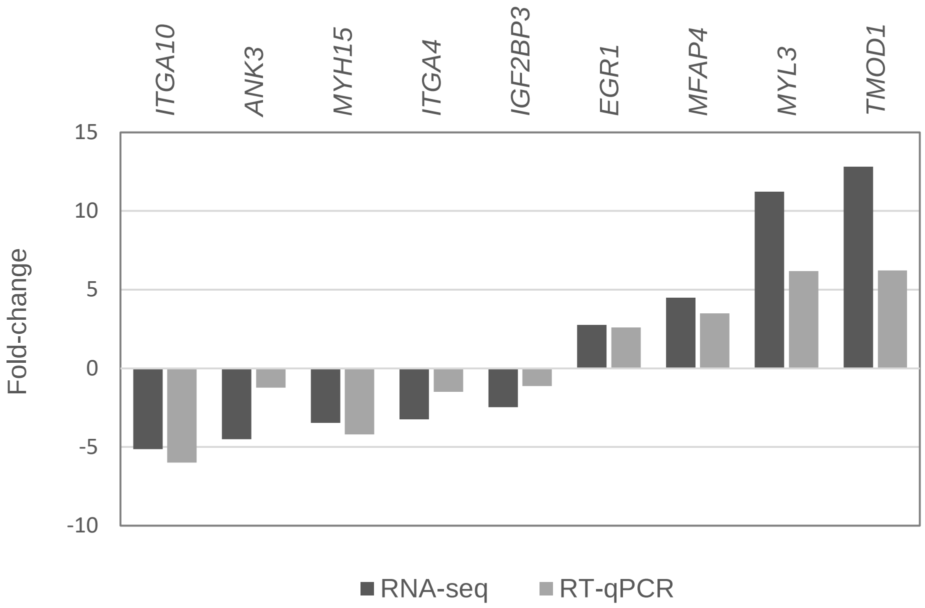

Supplementary Fig. 1. Real time quantitative PCR (RT-qPCR) validation of the discovery cohort array at high glucose. Pearson correlation coefficient of r=0.95 between RNA-seq and RT-qPCR (P-value=0.00019).
