# Supplementary material for: Transcriptome analysis in LRRK2 and idiopathic Parkinson’s disease at different glucose levels
Source: NPJ Parkinsons Dis. 2021 Dec 1;7:109. doi: 10.1038/s41531-021-00255-x (PMC8636510; doi:10.1038/s41531-021-00255-x)
Supplement: Supplementary file 5 — Reporting Summary [file 41531_2021_255_MOESM5_ESM.pdf]

nature portfolio

Corresponding author(s): NPJPARKD-00804R

Last updated by author(s): Sep 21, 2021

Reporting Summary

Nature Portfolio wishes to improve the reproducibility of the work that we publish. This form provides structure for consistency and transparency in reporting. For further information on Nature Portfolio policies, see our Editorial Policies and the Editorial Policy Checklist.

Statistics

For all statistical analyses, confirm that the following items are present in the figure legend, table legend, main text, or Methods section.

n/a	Confirmed	
☐	☒	The exact sample size (*n*) for each experimental group/condition, given as a discrete number and unit of measurement
☐	☒	A statement on whether measurements were taken from distinct samples or whether the same sample was measured repeatedly
☐	☒	The statistical test(s) used AND whether they are one- or two-sided<br>*Only common tests should be described solely by name; describe more complex techniques in the Methods section.*
☐	☒	A description of all covariates tested
☐	☒	A description of any assumptions or corrections, such as tests of normality and adjustment for multiple comparisons
☐	☒	A full description of the statistical parameters including central tendency (e.g. means) or other basic estimates (e.g. regression coefficient) AND variation (e.g. standard deviation) or associated estimates of uncertainty (e.g. confidence intervals)
☐	☒	For null hypothesis testing, the test statistic (e.g. *F*, *t*, *r*) with confidence intervals, effect sizes, degrees of freedom and *P* value noted<br>*Give P values as exact values whenever suitable.*
☒	☐	For Bayesian analysis, information on the choice of priors and Markov chain Monte Carlo settings
☐	☒	For hierarchical and complex designs, identification of the appropriate level for tests and full reporting of outcomes
☐	☒	Estimates of effect sizes (e.g. Cohen's *d*, Pearson's *r*), indicating how they were calculated

Our web collection on statistics for biologists contains articles on many of the points above.

Software and code

Policy information about availability of computer code

Data collection	software Real Time Analysis (RTA 1.18.61)
Data analysis	STAR software version 2.5.1b, GENCODE version 24, Clustvis software.

For manuscripts utilizing custom algorithms or software that are central to the research but not yet described in published literature, software must be made available to editors and reviewers. We strongly encourage code deposition in a community repository (e.g. GitHub). See the Nature Portfolio guidelines for submitting code & software for further information.

Data

Policy information about availability of data

All manuscripts must include a data availability statement. This statement should provide the following information, where applicable:

- Accession codes, unique identifiers, or web links for publicly available datasets
- A description of any restrictions on data availability
- For clinical datasets or third party data, please ensure that the statement adheres to our policy

Data availability
GEO data of the study and study details are deposited in the publicly available repositories GSE82340 and GSE167529.

Field-specific reporting

Please select the one below that is the best fit for your research. If you are not sure, read the appropriate sections before making your selection.

☒ Life sciences ☐ Behavioural & social sciences ☐ Ecological, evolutionary & environmental sciences

For a reference copy of the document with all sections, see nature.com/documents/nr-reporting-summary-flat.pdf

Life sciences study design

All studies must disclose on these points even when the disclosure is negative.

Sample size	No specific sample size calculation was performed but we used similar number of fibroblast lines to other previous transcriptomic studies in PD with subsequent replication of findings.
Data exclusions	No data were excluded, not outliers were observed nor excluded. Inclusion criteria included patients with idiopathic PD and LRRK2-associated PD.
Replication	We performed a pilot study and a subsequent validation study (at two different glucose concentration conditions). The validation study confirmed the presence of gene enrichment for specific gene ontology terms.
Randomization	Allocation of fibroblast lines in the study was random but assuring age and gender matching between PD and controls.
Blinding	Allocation and identification of sample types was blinded to operator during RNA-seq data collection. In addition, all PD and controls of each study were analysed in the same RNA-seq batch. Subsequent statistical analysis of the resulting data was performed in automatized platforms not affected by blinding.

Reporting for specific materials, systems and methods

We require information from authors about some types of materials, experimental systems and methods used in many studies. Here, indicate whether each material, system or method listed is relevant to your study. If you are not sure if a list item applies to your research, read the appropriate section before selecting a response.

Materials & experimental systems

n/a	Involved in the study	
☒	☐	Antibodies
☐	☒	Eukaryotic cell lines
☒	☐	Palaeontology and archaeology
☒	☐	Animals and other organisms
☐	☒	Human research participants
☒	☐	Clinical data
☒	☐	Dual use research of concern

Methods

n/a	Involved in the study	
☒	☐	ChIP-seq
☒	☐	Flow cytometry
☒	☐	MRI-based neuroimaging

Eukaryotic cell lines

Policy information about cell lines

Cell line source(s)	Each cell line (fribroblasts from primary skin biopsies) was obtained from our own collected subjects. These cell lines are stored in our official biorepository code C.0000253 (Instituto de Salud Carlos III, Spain).
Authentication	Cell lines were not authenticated. However, we have stored blood samples from the same individuals to allow possible future authentication (official collection registered at the Spanish ISCIII). These cell lines are stored for future research.
Mycoplasma contamination	All cell lines were negative for mycoplasma contamination.
Commonly misidentified lines (See ICLAC register)	We did not use commercial lines (individual codes showed at Table 1)

Human research participants

Policy information about studies involving human research participants

Population characteristics	All relevant characteristics are described in Table 1
Recruitment	Primary skin biopsies were obtained from iPD patients without a family history of disease and without LRRK2 mutations, L2PD

Recruitment	patients carrying the G2019S mutation, and cultured fresh. Fibroblast of healthy controls were obtained from spouses of the patients. From our fibroblast biorepository we further selected random PD patients; and then controls with similar age and gender percentages (discovery study), or age and gender matched controls (validation study).
Ethics oversight	The Ethics committee of the Hospital Clinic of Barcelona approved the study.

Note that full information on the approval of the study protocol must also be provided in the manuscript.
